# First Genomic Characterization of *bla*_VIM-1_ and *mcr-9-*Coharbouring *Enterobacter* *hormaechei* Isolated from Food of Animal Origin

**DOI:** 10.3390/pathogens9090687

**Published:** 2020-08-22

**Authors:** Mustafa Sadek, Hirofumi Nariya, Toshi Shimamoto, Shizuo Kayama, Liansheng Yu, Junzo Hisatsune, Motoyuki Sugai, Patrice Nordmann, Laurent Poirel, Tadashi Shimamoto

**Affiliations:** 1 Medical and Molecular Microbiology, Faculty of Science and Medicine, University of Fribourg, 1700 Fribourg, Switzerland; patrice.nordmann@unifr.ch (P.N.); laurent.poirel@unifr.ch (L.P.); 2 Food Hygiene and Control, Faculty of Veterinary Medicine, South Valley University, Qena 83511, Egypt; 3 Laboratory of Food Microbiology and Hygiene, Graduate School of Integrated Sciences for Life, Hiroshima University, Higashi-Hiroshima 739-8528, Japan; nariya@jumonji-u.ac.jp (H.N.); tsima@hiroshima-u.ac.jp (T.S.); 4 Antimicrobial Resistance Research Center, National Institute of Infectious Diseases, Higashimurayama 189-0002, Japan; kayama@niid.go.jp (S.K.); yu@niid.go.jp (L.Y.); hisatune@niid.go.jp (J.H.); sugai@niid.go.jp (M.S.); 5 Swiss National Reference Center for Emerging Antibiotic Resistance (NARA), University of Fribourg, 1700 Fribourg, Switzerland; 6 INSERM European Unit (IAME, France), University of Fribourg, 1700 Fribourg, Switzerland; 7 Institute for Microbiology, University of Lausanne and University Hospital Centre, 1011 Lausanne, Switzerland

**Keywords:** *Enterobacter cloacae* complex, *mcr-9*, VIM-1, IncHI2, WGS, Egypt, food

## Abstract

We describe here the complete genome sequence of an *Enterobacter hormaechei* ST279 coharbouring *bla*_VIM-1_ and *mcr-9* recovered from uncooked beef patty in June 2017, Egypt. The tested isolate was resistant to carbapenem but susceptible to colistin (minimum inhibitory concentration (MIC), 0.5 μg/mL). The antimicrobial susceptibility profile and conjugation experiments were performed. The entire genome was sequenced by the Illumina MiniSeq and Oxford Nanopore methods. The *bla*_VIM-1_ and *mcr-9* genes are carried on the same IncHI2/pMLST1 plasmid, pMS37a (Size of 270.9 kb). The *mcr-9* gene was located within the physical boundaries demarcated by two insertion elements IS*903* (upstream) and IS*1* (downstream) but did not possess the downstream regulatory genes (*qseC*/*qseB*) which regulate the expression of *mcr-9*. Therefore, the *mcr-9* might be silently disseminated among carbapenem-resistant Enterobacterales. In addition to *bla*_VIM-1_ and *mcr-9*, plasmid pMS37a harbored various antibiotic resistance genes including *aac(6’)-Il*, Δ*aadA22*, *aac(6’)-Ib-cr*, *sul1*, *dfrA1 and tetA.* To the best of our knowledge, this is the first report of a *bla*_VIM-1_ and *mcr-9*-coharbouring *E. hormaechei* isolate of food origin worldwide. The identification of a multidrug-resistant VIM-1 and *mcr-9* positive *Enterobacter hormaechei* isolate from food is worrisome as retail meat and meat products could serve as a vehicle for these MDR bacteria, which could be transferred between animals and humans through the food chain. It further highlights that Enterobacterales co-producing MCR and carbapenemases being found in the food chain indeed correspond to a One-Health issue, highlighting the need for serious steps to prevent their further dissemination.

## 1. Introduction

Carbapenemase-producing Enterobacterales (CRE) are one of the most clinically serious multidrug-resistant (MDR) bacteria in medical healthcare worldwide [1]. CRE infections are associated with high rate of morbidity and mortality due to the limited availability of therapeutic choices and the lack of development of new antimicrobial agents [2]. The major five carbapenemases identified in clinical isolates are *Klebsiella pneumoniae* Carbapenemase (KPC) enzymes, New Delhi Metallo-β-lactamase (NDM), Verona Integron-encoded Metallo-β-lactamase (VIM), IMiPenemase (IMP) enzymes, and oxacillinase (OXA-48) and its derivatives [3,4]. VIM-producing Enterobacterales have been reported in several countries, especially in the Mediterranean region including Kuwait, the United Arab Emirates, and Egypt [5,6,7]. Polymyxins are considered as the last line of defense against serious clinical infections caused by carbapenem-resistant Gram-negative bacteria, especially CRE [8]. Until recently, colistin resistance was mainly linked to deletion(s) or mutation(s) of two component systems (*phoPQ* and *pmrAB*) involved in the biosynthesis of the lipopolysaccharide [9]. However, concerns were raised since the first report of a new plasmid-mediated colistin resistance mechanism, MCR-1, which has been detected in China in late 2015 from the environment, food, animals and humans [10]. Very recently, a novel *mcr* homologue, *mcr-9*, harbored by a *Salmonella enterica* serotype Typhimurium isolate was retrieved from an American patient [11]. Infections caused by multidrug resistant isolates coproducing MCR and carbapenemases may result in significant clinical and public health concerns, as treatment options in such cases are very limited. Here, we report a multidrug-resistant *Enterobacter hormaechei* strain, of food origin, coharbouring the *bla*_VIM-1_ and *mcr-9* genes from Egypt. The genetic characteristics of this strain were analyzed using both of the Illumina and Oxford Nanopore DNA sequencing platforms. The whole-genome sequence (WGS) analysis identified *mcr*-9 gene located on the same IncHI2 plasmid harbouring *bla*_VIM-1_. To the best of our knowledge, this is the first global report of an *E. hormaechei* isolate of food origin coharbouring *bla*_VIM-1_ and *mcr-9*.

The *E. hormaechei* MS37 isolate was recovered from uncooked beef patty in Egypt in 2017. The API 20E System (BioMerieux) and the sequencing of the 16S ribosomal RNA gene identified the strain MS37 as *E. cloacae* complex that later was identified as *E. hormaechei* by WGS. The minimal inhibitory concentrations (MICs) were determined using the broth microdilution according to the European Committee on Antimicrobial Susceptibility Testing (EUCAST) (www.eucast.org) and Clinical and Laboratory Standards Institute (CLSI) guidelines (Document, M100-S24). The strain MS37 was resistant to ampicillin (MIC, >128 μg/mL), ceftazidime (MIC, >128 μg/mL), cefotaxime (MIC, >128 μg/mL), ceftriaxone (MIC, >128 μg/mL), meropenem (MIC, >32 μg/mL), ertapenem (MIC, >32 μg/mL), imipenem (MIC, >32 μg/mL), ceftazidime-avibactam (MIC, >32 μg/mL), kanamycin (MIC, >32 μg/mL), fosfomycin (MIC, >32 μg/mL), and tetracycline (MIC, 16 μg/mL), but was still susceptible to aztreonam (MIC, 0.25 μg/mL), and colistin (MIC, 0.5 μg/mL). The phenotypic carbapenemase production was confirmed using the Carba NP test [12]. Polymerase chain reaction (PCR) and DNA sequencing were used to detect the following carbapenem-hydrolyzing enzyme encoding genes: *bla*_VIM_*, bla*_KPC_, *bla*_IMP_, *bla*_NDM_, *bla*_OXA-48-like_, *bla*_BIC_, *bla*_AIM_, *bla*_SPM_, *bla*_DIM_, *bla*_GIM_, and *bla*_SIM_ as described previously [13]. The isolate was positive for the *bla*_VIM-1_ gene. A conjugation experiment was done using the MS37 strain as donor and the *E. coli* J53 strain (F^−^ *met pro* Azi^r^) as recipient. Transconjugants were selected on LB agar plates supplemented with ampicillin (100 µg/mL) and sodium azide (150 µg/mL) at 25–30 ℃. Results showed that the *bla*_VIM-1_ and *mcr-9*-coharbouring plasmid was successfully transferred to the recipient *E. coli* J53 [14].

## 2. Materials and Methods

For further understanding of the genetic environment of the *bla*_VIM-1_-harbouring plasmid and to analyze all the plasmids contained in that strain, we used both of the short- and long-read methods for the genome sequencing of MS37 strain. The total genomic DNA (gDNA) was extracted from an overnight bacterial culture of strain MS37 strain using the Qiagen Genomic-tip 20/G kit (Qiagen, Tokyo, Japan). WGS was done using a combination of the Illumina MiniSeq (Illumina, San Diego, CA, USA) and MinION (Oxford Nanopore Technologies, Oxford, UK) platforms. For MiniSeq sequencing, a DNA library was constructed using the Nextera XT Library Prep Kit and Nextera XT Index Kit (Illumina, San Diego, CA, USA) according to the manufacturer’s instructions. For MinION sequencing, library preparation was performed using the SQK-RBK004 Rapid Barcoding Kit and the DNA was subsequently sequenced using a FLO-MIN106 (R9.4) flow cell from Oxford Nanopore Technologies (ONT). Hybrid assembly of both Illumina short reads and Nanopore long reads were performed using the Unicycler assembly pipeline [15]. Hybrid assembly generated a complete chromosome of 4,673,152 bp and 4 plasmids ranging in size from 6851 bp to 270,915 bp (Table 1). The plasmid sequence was automatically annotated using the RAST server using the RAST-tk scheme [16], followed by manual inspection and correction using the BLASTn and BLASTp programs (https://blast.ncbi.nlm.nih.gov/Blast.cgi) using a 99% identity cutoff. Multilocus sequence typing (MLST) analysis (https://cge.cbs.dtu.dk/services/MLST/) showed that strain MS37 belonged to sequence type (ST) ST279 (allelic profile 67-20-19-45-45-4-32). Antimicrobial resistance genes were determined by ResFinder 3.2 (https://cge.cbs.dtu.dk/services/ResFinder/) and RGI (https://card.mcmaster.ca/analyze/rgi). The chromosome of MS37 harbored *bla*_ACT-16_, an intrinsic AmpC enzyme of *E. cloacae* complex species, which confer resistance to some β-lactams and *fosA* which confer resistance to fosfomycin. The plasmid incompatibility groups and pMLST were identified by PlasmidFinder 2.1 (https://cge.cbs.dtu.dk/services/PlasmidFinder/) and pMLST 2.0 (https://cge.cbs.dtu.dk/services/pMLST/), respectively. Ethical Approval: Not required.

## 3. Results

The isolate harbored a total of four plasmids (pMS37a, pMS37b, pMS37c, and pMS37d) belonging to incompatibility groups IncHI2/IncHI2A (pMLST1), IncC, IncFIB and ColRNAI (Table 1). In addition to *bla*_VIM-1_, plasmid pMS37a harbored other antibiotic resistance genes including genes encoding resistance to aminoglycosides (*aac(6’)-Il*, *ΔaadA22*), aminoglycosides and fluoroquinolones (*aac(6’)-Ib-cr*), sulfonamides (*sul1*), trimethoprim (*dfrA1*), and tetracycline (*tetA*). No antibiotic-resistance genes were detected on the other plasmids (pMS37b, pMS37c, and pMS37d).

**Figure 1:**
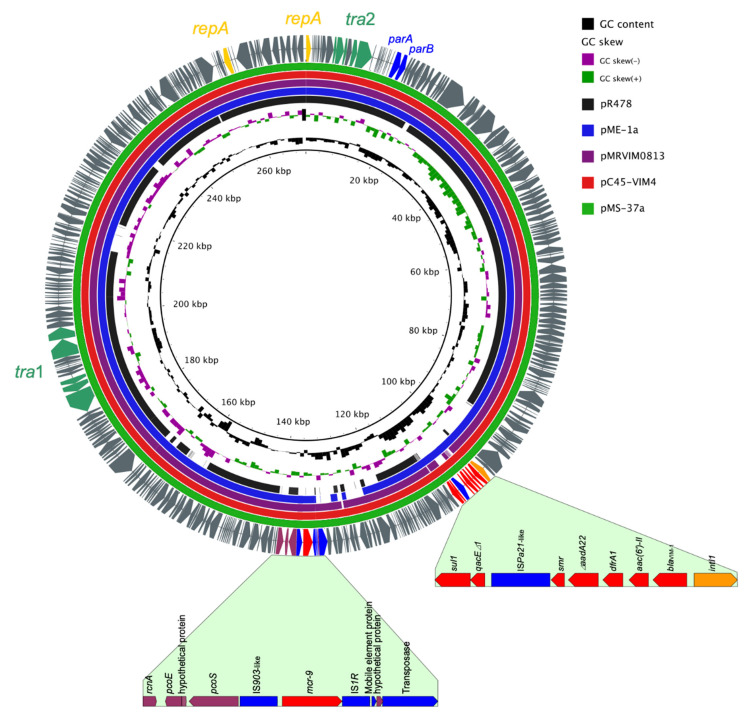
Circular map of *bla*_VIM-1_ and *mcr-9*-coharbouring IncHI2 plasmid compared to other reported similar plasmids. The complete sequence of pMS37a (the outer circle) was used as a reference plasmid. The circular maps were generated using the BRIG software and plasmids were included in the following order (inner to outer circles): pR478 (GenBank ID: BX664015.1), pME-1a (CP041734), pMRVIM0813 (KP975077), pC45-VIM4 (LT991958), and pMS37a (this study, accession no. CP053191). The resistance loci were highlighted in full (the gene cassette arrays of class 1 integron and the genetic structure surrounding the *mcr-9* gene). The different colors indicate different plasmids and are listed in the color key.

Surprisingly, the plasmid pMS37a was found to harbor the recently reported *mcr-9* (colistin resistance gene), identical to the *mcr-9* gene (100% query coverage and 100% sequence identity) firstly identified in a *Salmonella* isolate [11]. Analysis of WGS data of *E. hormaechei* MS37 showed that *bla*_VIM-1_ and *mcr*-9 genes were located on an IncHI2 incompatibility group plasmid, 270,915 bp in size with an average G+C content of 46% (Table 1 and Figure 1). PCR-based replicon typing (PBRT) analysis and alkaline lysis performed from isolate MS37, followed by gel electrophoresis analysis, confirmed the result of WGS. IncHI2-ST1 plasmids are large (>250 kb) conjugative plasmids with a broad host range. IncHI2-plasmids, serving as a critical reservoir of genes conferring resistance to critically important antibiotics and heavy metals among Enterobacterales in the Middle East and Africa, have been reported previously from humans, companion animals, chicken, food and pigs [17,18,19,20,21].

The circular image and circular comparisons between other reported similar plasmids were done using the BRIG tool (Figure 1) [22]. The sequence of plasmid pMS37a was highly similar to that of the VIM-4 and *mcr-9* positive-IncHI2 plasmid, pME-1a (97% query coverage and 100% sequence identity; GenBank accession no. CP041734.1) identified, surprisingly, from an *E. hormaechei* isolate recovered from an American patient, with a history of travel to Egypt, who had a throat infection in Egypt [23]. The plasmid pME-1a was found to contain a novel integron with the cassette array of *dfrA16*-*aadA2*-*dfrA16*-*aadA2*-*smr* which is absent in our plasmid [23]. Also, contrarily to pME-1a, plasmid pMS37a did not appear to harbor the extended-spectrum β-lactamase (ESBL) bla_CTX-M-9_ [23]. Also, this plasmid pMS37a was highly similar to other IncHI2 plasmids e.g. pC45-VIM4 (100% query coverage and 99.98% sequence identity; GenBank accession number LT991958) and pMRVIM0813 (98% query coverage and 99.99% sequence identity; GenBank accession number KP975077.1) (Figure 1). Compared to the IncHI2 prototype plasmid R478 (GenBank accession number BX664015.1), the common regions mainly encode core plasmid determinants, which might define the general IncHI2 backbone (i.e., replication, maintenance, stability, and transfer systems). The conjugative transfer genes in pMS37a are distantly locating in two separate sites similar to plasmid R478 (*Tra1* and *Tra2*), presumably facilitating the spread of this plasmid between various members of Enterobacterales.

## 4. Discussion

The *bla*_VIM-1_ gene was located in a class 1 integron with the cassette array of *int1*-*bla*_VIM-1_-*aac(6’)-Il*-*dfrA1*-*ΔaadA22*-*smr*-IS*Pa21_-like_*-*qacEΔ1*-*sul1.* Further analysis of the genomic data from *E. hormaechei* MS37 revealed that *mcr-9* gene was located within the physical boundaries demarcated by two insertion elements IS*903* (upstream) and IS*1* (downstream), similar structures to those of pMRVIM0813 (accession no. KP975077), pCTXM9_020038 (accession no. CP031724), and pME-1a (accession no. CP041734) with all of them lacking the *qseB* and *qseC* regulatory genes. A different genetic structure surrounding the *mcr-9* gene has been detected from other IncHI2 plasmids (*qseB*-*qseC*-*wbuC*-*mcr-9*-IS*903*), e.g., pT5282-mphA (accession no. KY270852) and pN1863-HI2 (accession no. MF344583). The genetic context of *mcr-9* identified in our study lacks a two-component regulatory system encoded by the *qseC* and *qseB* genes which is very important in the induction of colistin resistance mediated by *mcr-9* [24]. This could explain the susceptibility of the *E. hormaechei* MS37 described here to colistin (MIC, 0.5 μg/mL). In contrast, a recent study identified a clinical *E. hormaechei* isolate from China carrying *qseC* and *qseB* downstream of *mcr-9* and resistant to colistin (MIC of 16 μg/mL), demonstrating the importance of this two-component systems for expression of this phenotype [25]. The *mcr-9* gene may be silently spreading in Enterobacterales throughout the world and because of that, the prevalence of *mcr-9* is unclear especially this gene is not actually related to colistin resistance, raising the likelihood of ongoing undetected dissemination [24].

## 5. Conclusions

In conclusion, we described here for the first time the complete genomic characterization of *bla*_VIM-1_ and *mcr-9*-coharbouring *E. hormaechei* isolate of food origin. In fact, the identification of a multidrug-resistant VIM-1 and *mcr-9* positive isolate, *E. hormaechei*, from food is worrisome as retail meat and meat products could serve as a vehicle for these MDR bacteria, which could be transferred between animals and humans through the food chain. It further highlights that Enterobacterales coproducing MCR and carbapenemases may be a true One-Health issue and close epidemiologic surveillance is urgently needed to control their further spread.

The complete genome sequence of MS37 strain and all plasmids had been deposited at GenBank under BioProject ID: PRJNA630061. The complete nucleotide sequences of the chromosome of MS37, pMS37a, pMS37b, pMS37c, and pMS37d were deposited as GenBank accession numbers CP053190, CP053191, CP053192, CP053193, and CP053194, respectively.

## Figures and Tables

**Table 1:** Characteristics of chromosome and plasmids harbored by *E. hormaechei* MS37.

Genetic Element	Size (bp)	MLST	pMLST	Plasmid Incompatibility Group	Antibiotic Resistance Gene(s)
**Chromosome**	4,673,152	ST279	NA	NA	*fosA*, *bla*_ACT-16_
**pMS37a**	270,915	NA	ST1	IncHI2/IncHI2A	*sul1*, *mcr*-9, *bla*_VIM-1_, *tet(A)*, *aac(6’)-Ib-cr*, *aac(6’)-Il,* Δ*aadA22*, *dfrA1*
**pMS37b**	129,016	NA	Unknown	IncC	NA
**pMS37c**	108,277	NA	Unknown	IncFIB	NA
**pMS37d**	6851	NA	Unknown	ColRNAI	NA

NA, not applicable.
